# Assessment of the effects of a multi-component, individualized physiotherapy program in patients receiving hospice services in the home

**DOI:** 10.1186/s12904-020-00600-6

**Published:** 2020-07-09

**Authors:** Agnieszka Ćwirlej-Sozańska, Agnieszka Wójcicka, Edyta Kluska, Anna Stachoń, Anna Żmuda

**Affiliations:** Institute of Health Sciences, Medical College of Rzeszow University, Rzeszow, Poland

**Keywords:** Hospice, Disability, Physiotherapy, ADL, Balance, Quality of life, ICF

## Abstract

**Background:**

The interest in physiotherapy programs for individuals in hospice is increasing. The aim of our study was to assess the impact of a multi-component, individualized physiotherapy program on the functional and emotional conditions and quality of life of patients receiving hospice services in the home.

**Methods:**

The study included 60 patients (mean 66.3 years) receiving hospice services in the home. A model of a physiotherapy program was designed, including breathing, strengthening, transfer, gait, balance, functional, and ergonomic exercises, as well as an adaptation of the patient’s living environment to functional needs. The tests were performed before and after the intervention. The study used the Activities of Daily Living (ADL) and Instrumental Activities of Daily Living (IADL) scales, the World Health Organization Quality of Life - Bref (WHOQOL-BREF), the Visual Analogue Scale (VAS) pain scale, the Tinetti POMA Scale, and the Geriatric Depression Scale (GDS). To enable comparison of our results worldwide, a set of International Classification of Functioning, Disability and Health (ICF) categories was used.

**Results:**

The average functional level of the ADL (mean 2.9) and the IADL (mean 11.9), as well as the WHOQOL-BREF (mean 46.4) of the patients before the intervention were low, whereas the intensity of pain (VAS mean 5.8), the risk of falling (Tinetti mean 8.2), and depression (GDS mean 16.7) were recorded as high. After the completion of the intervention program, a significant improvement was found in the ADL (mean 4.0), IADL (mean 13.9), WHOQOL-BREF (mean 52.6), VAS (mean 5.1), risk of falling (Tinetti mean 12.3), and GDS (mean 15.7) scores.

**Conclusions:**

The physiotherapeutic intervention had a significant impact on improving the performance of ADL, as well as the emotional state and quality of life of patients receiving hospice services in the home. The results of our research provide evidence of the growing need for physiotherapy in individuals in hospice and for comprehensive assessment by means of ICF.

Registered 02.12.2009 in the Research Registry (https://www.researchregistry.com/why-register) under the number research registry 5264.

## Background

In recent years, there has been an increased interest in the use of physiotherapy in patients cared for in palliative care [1]. The goal of physiotherapy in this group of patients is to minimize the negative effects of the disease or invasive treatment. Rehabilitation in palliative care must be individualized and it should include various actions, such as a training of mobility, transfer, and balance, a program improving respiratory functions, a lymphoedema therapy, strengthening exercises, pain relief programs, education, and psychological support [2]. Initially, physiotherapy was aimed at providing general support to hospice patients. However, there is more evidence emerging nowadays that highlights the positive and relatively persistent effects of physiotherapy in the discussed group of patients. It is worth mentioning that physical exercises can reduce pain in palliative patients [3]. Moreover, these exercises can improve strength, endurance, and function, or slow down the decline in individuals in hospice [4]. They can enhance respiratory functions and performance while performing daily activities [5]. Some studies indicate that in this group of patients, physiotherapeutic interventions improve the quality of life [6], decrease pain and anxiety [7, 8]. However, there are very few studies assessing the effects of physiotherapy programs in hospices [6]. Assessments of functional performance and quality of life are considered to be a measure of the effectiveness of palliative care [9, 10].

Disability is a common problem among individuals in hospice [11]; this group of patients is characterized by a very large diversity in terms of health, functioning, and disability, as well as a subjective assessment of the quality of life. Moreover, many patients in hospices are diagnosed with depression [12].

The use of a physiotherapy model based on the biopsychosocial model of the International Classification of Functioning, Disability and Health (ICF) of the World Health Organization (WHO) in this group of patients seems to be an interesting solution [13]. This model provides a multidimensional approach to functioning and disability. Disability is a broad concept including impairment in body function, activity restrictions as well as participation restrictions. It defines the negative aspects of the interaction between individuals with a specific medical condition and environmental and personal factors. Disability perceived in this way is not strictly assigned to a specific disease entity [14]. An initial assessment and evaluation are based on ICF codes, to which appropriately selected measurement tools are linked. Rehabilitation goals are set in collaboration with the patient with the aim to address his/her needs and expectations [15]. The application of the ICF gives the opportunity to comprehensively assess patients’ problems and needs, as well as to present research results in an accessible and comparable manner by various researchers [16].

To the best of our knowledge, very little research has been carried out thus far to assess the impact of physiotherapy on the functioning of patients receiving hospice services in the home, or on the effectiveness and usefulness of physiotherapy programs in hospice care. There are also no reports on the assessment of physiotherapy in individuals in hospice by means of ICF [17].

The aim of the study was to assess the impact of a multi-component, individualized physiotherapy program on the functional and emotion states, as well as quality of life of patients receiving hospice services in the home. The impact of the physiotherapy program on performing basic daily activities (Activities of Daily Living; ADL) in patients with hospice services in the home was indicated as the primary objective. The secondary objectives were: the impact of the physiotherapy program on the performance of complex daily activities (Instrumental Activities of Daily Living; IADL), mobility and balance (Tinetti POMA Scale), emotional state (Geriatric Depression Scale; GDS), and quality of life (World Health Organization Quality of Life; WHOQOL-BREF) in patients receiving hospice services in the home.

## Methods

### Study design

The study was a one group pre-test post-test design where data were collected before and after an intervention containing a multi-component, individualized physiotherapy program on patients referred for hospice care in the home in the period from March to June 2019.

### Participants

The study included 60 patients aged 55–89 with hospice services in the home, living in southern Poland (Małopolskie and Podkarpackie Voivodships). Participants were recruited from two of the rehabilitation centers participating in the study. Patients or family reporting to the center to receive a rehabilitation services in the home were informed about the possibility of participating in the project. After the patient’s attending physician referred them to the hospice system, the hospice center’s physician evaluated the patient’s health and functional state. If appropriate, the hospice physician referred them to multi-component, individualized physiotherapy program. Patients with better prognosis as to the length of the survival time and who were not terminal stage participated in the program. The inclusion criteria of the project were: age ≥ 55, normal cognitive state (Abbreviated Mental Test Score; AMTS > 6 points) [18], and a patient’s informed consent to participate in the study. The exclusion criteria were: age < 55, cognitive state ≤6 points on the AMTS scale or unconsciousness, terminal stage (close to death), and lack of informed consent of the subjects to participate in the program. The examination and physiotherapy program were performed at the place of residence of the participants by specialized physiotherapists.

**Fig. 1:**
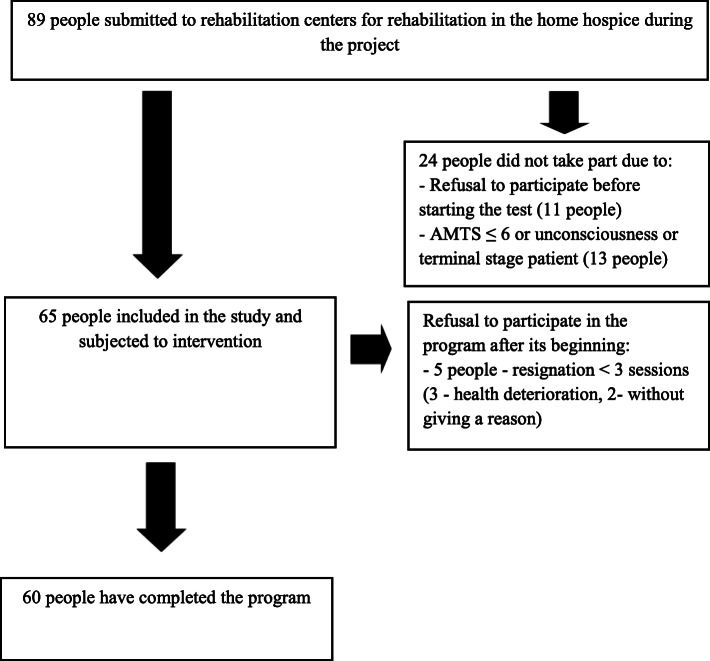
Flow diagram of the study

All consecutively admitted patients from March to June 2019 who met the inclusion criteria were qualified for the program. Overall, 65 patients were qualified for program participation, eventually 60 patients took part (Fig. 1). The short duration of the project and its pilot nature influenced the decision (supported by ethical considerations) to create one study group and to deliver support to all patients reported to the project.

### Setting

The assessment of the functional and emotional states and the quality of life of patients was carried out twice: before the beginning of the physiotherapy program (pre-test) and after the end of the program (post-test). A quasi-blind test was used, in which one physiotherapist performed the pre- and post-tests while another specialist carried out the physiotherapy program. All physiotherapists implemented improvements in accordance with the model of the multi-component, individualized physiotherapy program developed in the project (Table 1). Both the pre-test and post-test, as well as the physiotherapy program, took place in the morning in the patient’s home.

**Table 1:** A model of the multi-component individualized physiotherapy program for patients receiving hospice service in the home

A model of the multi-component individualized physiotherapy program	
1. Mental and physical preparation of the patient for exercises - greetings, questions about well-being, motivational conversation - encouragement for doing exercises together (determining together closer and further objectives of improvement). 2. Breathing exercises in a lying or sitting position. 3. Breathing exercises together with active or supporting exercises for the upper and lower limbs in a lying and sitting position. Exercises for major muscle groups. The number of repetitions and sets are tailored to the patient’s condition (on average 2–3 sets of 4–8 repetitions, during the first session the number of sets is 1 and it is increasing during the program if the patient’s condition allows it) 4. Patient’s standing up - sitting down with or without a support, standing up. 5. Transfer exercises - moving from the bed to the chair/armchair and coming back. 6. Walking exercises - walking on a flat ground, walking on a variable ground (carpet, thresholds, etc.) 7. Balance exercises in sitting and/or standing position with an assistance of the physiotherapist. 8. Exercises using home equipment (furniture, walls, handles, etc.) to move around the house safely. 9. Exercises using auxiliary equipment (walking stick, crutch, walker) to move around the house. 10. Ergonomic exercises in walking, lifting and moving objects, performing everyday activities. 11. Advice on the reorganization of home space in order to adapt it to the functional needs of the patient. 12. Education in the field of safety rules when moving inside and outside the home (if possible). Ways of dealing with dizziness, weakness, etc. while moving or performing daily activities. 13. Learning and training to be aware of benefits associated with rest and relaxation. 14. Education of the patient’s family after each session in the scope of performing exercises with the patient on days when sessions with the physiotherapist were not carried out.	
Individual points of the program are interspersed with breathing exercises and rest in a sitting or lying position.	
Each session is completed with recommendations for the patient and the carer on the patient’s activity between training sessions with a physiotherapist.	
The patient and the carer are encouraged to contact the physiotherapist or doctor by phone between sessions in case of any questions or concerns.	

The study was registered in the Research Registry under the number researchregistry5264.

### Intervention

The program was conducted for 6 weeks, twice a week (12 meetings in total). Each physiotherapy treatment session lasted, on average, 45 min and was performed by a physiotherapist. The program was based on the biopsychosocial model of the ICF [15].

In order to prepare the model of multi-component, individualized physiotherapy, the study was preceded by a review of international literature and it was based on the extensive clinical experience of a research team of physiotherapists working with hospice patients. The program was addressed to patients with better prognosis and with good cognitive status. An innovative element of the program was setting rehabilitation goals together with a patient receiving hospice services. Patients who can participate in making decisions about the goals of rehabilitation are more open to communication and ready to cooperate [19, 20]. The discussed program was focused on improving the patient’s functioning, both physically and mentally, with the particularly important areas being gait re-education, transfer training, active exercises, and strengthening the muscles. Cobbe and Kennedy indicated these areas as some of the most important in the rehabilitation of individuals in hospice [6]. Moreover, mental support and patients’ motivation for physical activity by physiotherapists is a very important area of the research. Burke et al. confirmed that interactions supporting and motivating terminally ill patients by healthcare professionals are an important factor facilitating involvement in physical activity [21]. Wiśniowska et al. demonstrated significantly better effects for physiotherapy programs associated with patient motivation techniques [22]. The novelty of the program was learning by the patients how to use elements of the environment to improve functioning and safety. Ergonomic exercises in getting around and lifting and moving objects during everyday activities were important. Another innovative element of the program was also the help of a physiotherapist in reorganizing the home space in order to adapt it to the functional needs of the patient. The patient’s environment may constitute a barrier or facilitation [23]; thus, eliminating barriers and maximizing facilitation allow the patients to get around better and to remain active in their environment [24]. Bethancourt et al. showed that structural barriers (e.g., inadequate equipment) could hinder the physical activity of people with reduced fitness [25]. Adapting the patient’s environment to provide a sense of safety and to minimize fear of falling, supporting balance and walking, could simulate movement, reduce physical discomfort, and encourage this person to be involved in activity and to independently participate in everyday life [26, 27].

An important element of the program was also learning to be aware of the benefits associated with rest and relaxation. It has been proven that relaxation of individual muscle groups involving muscle tension combined with deep breaths reduces pain and fatigue and improves the quality of life [28]. What is more, another essential part of the discussed program was the education of carers and their implementation in everyday work (physical and mental) with the patient in order to maximize his/her capabilities and to maintain the effects of physiotherapy. Training close relatives who care for chronically ill patients allows these patients to maintain the effects of physiotherapy in the area of patient activity and adjusting the living environment [25, 29].

The developed physiotherapy program was individually adapted to the patient’s health, age, and preferences. The model of this physiotherapy program is presented in Table 1.

### ICF assessment

A set of ICF categories was chosen for the selected areas within a range of functions, activities, and participation. Moreover, research tools were linked to the codes [30]. In accordance with the rules of ICF application, the selected codes were supplemented with qualifiers, indicating the severity of the health problem. A qualifier determined the size of the problem in the area described by the specific code. One qualifier was assigned to each code, in accordance with ICF generic scale [13]:

**Table:** 

xxx.**0** no problem	0–4%
xxx.**1** mild problem	5–24%
xxx.**2** moderate problem	25–49%
xxx.**3** severe problem	50–95%
xxx.**4** complete problem	96–100%
xxx.**8** not specified.	
xxx.**9** not applicable.	

### Data collection

Data were collected based on the patient’s interview (ADL, IADL, WHOQOL-BREF, GDS, VAS) and during the physical examination (Tinetti POMA Scale). The data with reference to ADL and IADL were confirmed by the relative or another caregiver responsible for the patient’s care. The average duration of the examination was 45 min. Information on the state of health and basic sociodemographic data were collected from the patient’s medical record. Due to the poor health and high fatigue of the studied patients, the number of research tools was minimized to the necessary minimum.

The following research tools were used for the study, which were linked to ICF codes:

1. Basic activities of daily living (ADL) – the Katz Index [31] containing 6 items, which were used to assess independence in the following activities: Bathing and showering (d510 Washing oneself), Dressing (d540 Dressing), Toilet hygiene (getting to the toilet, cleaning oneself, and getting back up) (d520 Caring for body parts and d530 Toileting), Transferring - functional mobility (d420 Transferring oneself), Self-feeding (not including cooking or chewing and swallowing) (d550 Eating), and controlled excretion of urine and stool (b525 Defecation functions and b620 Urination functions). The ADL scale was calculated in two variants - a standard scale and a modified scale, i.e., a 5-gradescale consistent with the scale of ICF problem assessment in order to assign easily qualifiers [13].
2. Instrumental Activities of Daily Living (IADL) - the Lawton Scale [32], containing 8 questions assessing independence in using the telephone or other forms of communication (d360 Using communication devices and techniques), moving further than walking distance (d470 Using transportation), shopping for groceries and necessities (d620 Acquisition of goods and services), preparing meals (d630 Preparing meals), cleaning and maintaining the house (d640 Doing housework), DIY/laundry (d650 Caring for household objects), taking prescribed medications (d598 Self-care, other specified, preparing and taking medication), and managing money (d860 Basic economic transactions).
  The IADL scale was calculated in two variants - a standard and a modified scale. As a standard scale, a 3-grade response scale was used for each item (1 = unable, 2 = needs assistance, 3 = independent) and sum the eight responses [33]. The modified scale included a 5-grade scale, consistent with the scale of ICF problem assessment in order to assign easily qualifiers [13].
3. WHOQO-BREF questionnaire for assessing the quality of life. This instrument contains 26 questions assigned to four domains assessing the quality of life related to health in the following areas: physical and psychological health, social relations, and the environment. Each question is rated on a 5-point scale. According to the WHO guidelines, the results for each domain are calculated by adding the values ​​of individual items, and then converted into a result in the range of 0–100, where 0 means the worst quality of life and 100 the highest quality of life [34]. ICF codes (b140 Attention functions, b130 Energy and drive functions, d920 Recreation and leisure, b134 Sleep functions, and d720 Complex interpersonal interactions) were linked to the selected WHOQOL-BREF items [30].
4. Geriatric Depression Scale (GDS). This tool allows the researcher to assess the patient’s emotional state and the occurrence of depressive moods. A 30-point scale was used. The participants received 0 or 1 point for each answer. The final result was the sum of the points. Interpretation of the results: 0–10 points, no depression; 11–20 points, mild depression; 21–30 points, deep depression [35]. ICF code b152 Emotional functions was linked with the GDS according to the ICF Linking Rules WHO [30].
5. Tinetti performance-oriented assessment of mobility problems (POMA) Scale, by which the function of dynamic balance and gait was assessed. Score scale: 26–28 points, no risk of falling; 19–25 points, moderate risk of falling; ≤18 points, 5 times higher risk of falling compared to people with a proper result [36, 37]. ICF codes d4106 Shifting body’s center of gravity and gait d450 Walking were linked with a relevant subscale assessing balance and gait according to the ICF Linking Rules WHO [30].
6. Visual Analogue Scale (VAS), assessing pain level - a simple and useful method for assessing the intensity of pain by marking a point on the line, e.g., 10 cm long, where 0 is attributed to the total absence of pain, and 10 the most severe pain imaginable. The ICF code (b280 Sensation of pain) was linked with VAS according to the ICF Linking Rules WHO [30].

### Statistical methods

The obtained data were analyzed using the Statistica TIBCO Software Inc. program (2017), Statistica (data analysis software system), version 13, http://statistica.io. For initial data analysis, descriptive statistics measures were used. The distribution for normality of features was tested by the Shapiro-Wilk test. The Wilcoxon test (measurable variables) and the McNemar test (non-measurable variables) were used to investigate the effect of therapy on changing the values of individual variables. The statistical significance was determined on the level of *p* < 0.05.

### Ethics approval

The study was approved by the Bioethical Commission of the University of Rzeszow (Decision number 2018/06/22b). All participants were familiarized with the purpose and the principles of the study, and informed about the possibility of withdrawing from the study at any stage. Before starting the research, the subjects signed their informed consent to participate in the study.

## Results

The average age of participants was 66.3 years. Most of the study group were men (53.3%) and persons living with a spouse or partner (60.0%) The majority of respondents lived with their family (95.0%). The main group of patients were patients with mainly gastrointestinal, lung, prostate, breast cancers (60.0%). Other patients suffered from neurological disease (23.3%; stroke, multiple sclerosis), or cardiovascular and metabolic diseases (16.7%; atherosclerosis, cardiomyopathy, diabetes) (Table 2).

**Table 2:** Characteristics of the study population (*n* = 60)

Variables		Total number (%) Mean (SD)
**Age**		66.3 (10.9)
**Gender**	Females	28 (46.7)
	Males	32 (53.3)
**Marital status**	Living with a spouse or partner	36 (60.0)
	Single	24 (40.0)
**Habitation**	With family	57 (95.0)
	Alone	3 (5.0)
**Primary disease**	Cancer including:	36 (60.0)
	• Gastrointestinal cancer	12 (20.0)
	• Respiratory system cancer	7 (11.7)
	• Breast cancer	7 (11.7)
	• Prostate cancer	7 (11.7)
	• Others	3 (5.0)
	Neurological disease including:	14 (23.3)
	• Ischemic stroke	8 (13.3)
	• Multiple sclerosis	6 (10.0)
	Cardiovascular or metabolic disease including:	10 (16.7)
	• Atherosclerosis	8 (13.3)
	• Cardiomyopathy	1 (3.2)
	• Diabetes	1 (3.2)

Abbreviation: *SD* standard deviation

The average functional level of ADL patients receiving hospice services in the home in the pre-test was 2.9 points out of a possible 6. Patients were varied in their disability level in performing basic everyday activities (SD = 2.2). After completion of the physiotherapeutic intervention, a significant improvement (*p* < 0.001) in average ADL was observed (4.0); however, group diversity remained significant (SD = 2.3). In the pre-test, the most common dependency was found in the performance of bathing and showering. Lack of independence in this area was observed in as many as 73.3% of the study population. After the intervention, the incidence of this problem statistically significantly decreased (53.3%; *p* = 0.002), but still, it was the most common problem among all of the basic activities of everyday life. Transferring - functional mobility was the second most common dependence in ADL. This problem in the pre-test was found in 61.7% of subjects; however, in the post-test, it occurred statistically significantly less frequently (*p* < 0.001) and was found in only 30.0% of the researched patients. Other activities that more than half of the subjects did not perform alone in the pre-test were dressing and toilet hygiene (getting to the toilet, cleaning oneself, and getting back up; 55.0% each). In both cases, a statistically significant improvement was observed after the intervention was completed (respectively, 30.0%, *p* = 0.001 and 36.7%, *p* = 0.003; Table 3).

**Table 3:** Assessment of the incidence of disability in individual items of ADLs and IADLs, before and after the intervention (*n* = 60)

Variables		Pre-test				Post-test				***P***-value
		Mean	SD	Me	Q	Mean	SD	Me	Q	
**ADL** including:		2.9	2.2	3.0	2.0	4.0	2.3	5.0	2.3	**< 0.001**^1^
		n (%)				n (%)				
	Bathing and showering	44 (73.3)				32 (53.3)				**0.002**^2^
	Dressing	33 (55.0)				18 (30.0)				**0.001**^2^
	Toilet hygiene	33 (55.0)				22 (36.7)				**0.003**^2^
	Transferring	37 (61.7)				18 (30.0)				**< 0.001**^2^
	Self-feeding	21 (35.0)				15 (25.0)				0.077^2^
	Controlled excretion of urine and stool	20 (33.0)				18 (30.0)				0.480^2^
**IADL**^**a**^ including:		11.9	4.4	10.0	3.3	13.9	5.0	14.0	4.8	**< 0.001**^1^
		n (%)				n (%)				
	Using the telephone or other form of communication	38 (63.3)				33 (55.0)				0.074^2^
	Moving further than a walking distance	58 (96.7)				51 (85.0)				**0.023**^2^
	Shopping for groceries and necessities	59 (98.3)				56 (93.3)				0.248^2^
	Preparing meals	55 (91.6)				48 (80.0)				**0.023**^2^
	Cleaning and maintaining the house	55 (91.7)				50 (83.3)				0.074^2^
	DIY/washing	58 (96.7)				54 (90.0)				0.134^2^
	Taking prescribed medications	48 (80.0)				42 (70.0)				**0.041**^2^
	Managing money	40 (66.7)				34 (56.7)				**0.041**^2^

Abbreviation: *SD* standard deviation, *Me* median, *Q* quartile deviation

^a^as a disability in individual IADL items; the necessity to obtain assistance in performing or lack of possibility to perform an activity was considered; ^1^Wilcoxon test; ^2^McNemar test

The average functional level in the IADL range of individuals was low - 11.9 in the pre-test out of a possible 24 points. In the post-test, a significant improvement (*p* < 0.001) was observed in the average IADL level (13.9), with a large group diversity (SD = 5.0). Shopping for groceries and necessities was the biggest problem, and this difficulty occurred in almost all patients before the physiotherapy program (98.3%). No significant improvement (*p* = 0.248) in this area was observed after completing the intervention, and it remained the area of the most common patient dependence. The next most frequently identified problems were moving further than walking distance and DIY/washing. In the pre-test, 96.7% of the study group had disability in this range. After the intervention, a considerable improvement (*p* = 0.023) was observed in the incidence of moving further than walking distance; however, it still occurred in the overwhelming majority of patients (85.0%). Additionally, there was no significant improvement in DIY/washing in the post-test study. However, improvement in independence after the physiotherapy program was observed in preparing meals, taking prescribed medications, and managing money (respectively, *p* = 0.023 and *p* = 0.041; Table 3).

In the pre-test, a high average pain intensity was found (5.8), with relatively low diversity in the group (SD = 1.8). After the intervention, a significant reduction (*p* < 0.001) in pain intensity was observed (5.1), although it remained mainly at a moderate level.

The pre-test study revealed that an average Tinetti POMA Scale result was low (8.2 points out of a possible 28) and indicated a high risk of falling. A significant improvement (p < 0.001) in the average Tinetti POMA Scale score (12.3) was observed in the post-test, as well as a significant improvement (*p* < 0.001) in both the Tinetti gait subscale assessing gait and the Tinetti balance subscale assessing balance. At the end of the program, the number of patients at high risk of falling decreased from 86.7 to 65.0%.

In the pre-test, depression was observed in all patients, including mild in 76.7% and deep in 23.3%. The average GDS score was 16.7 points. After the completion of the physiotherapy program, a significant improvement (*p* = 0.012) of the average GDS score was noted (15.7). The number of people with deep depression decreased from 23.3 to 18.3%.

The pre-test noted a low quality of life. The average number of points WHOQOL-BREF was below half the scale in the range of 0–100 (46.4). The lowest quality of life occurred in the psychological field (29.3), and the highest in the field assessing social relations (59.2). After completing the intervention, a significant improvement (*p* < 0.001) in the overall quality of life and the quality of life in each of the areas was found (Table 4).

**Table 4:** Assessment of parameters related to pain, risk of falling, balance and gait, emotional state and quality of life before and after intervention (*n* = 60)

Variables		Pre-test				Post-test				***P***-value
		Mean	SD	Me	Q	Mean	SD	Me	Q	
**Pain (VAS)**	Pain level on a scale of 0 to 10 point	5.8	1.8	5.0	0.8	5.1	1.8	4.0	1.0	**< 0.001**^1^
**Tinetti POMA Scale**	**Total (points)**	8.2	9.1	4.0	7.8	12.3	10.0	13.0	10.0	**< 0.001**^1^
	including:									
	No risk of falling (n, %)	5 (8.3)				9 (15.0)				
	Moderate risk of falling (n, %)	3 (5.0)				12 (20.0)				
	High risk of falling (n, %)	52 (86.7)				39 (65.0)				
	**Tinetti gait (points)**	3.8	4.4	1.0	3.8	5.4	4.9	5.5	5.0	**< 0.001**^1^
	**Tinetti balance (points)**	4.4	4.8	2.0	4.0	6.8	5.3	7.0	5.0	**< 0.001**^1^
**GDS**	**Total (points)**	16.7	4.0	16.0	2.8	15.7	4.0	16.0	3.0	**0.012**^1^
	including:									
	No depression (n, %)	0 (0.0)				1 (1.7)				
	Mild depression (n, %)	46 (76.7)				48 (80.0)				
	Deep depression (n, %)	14 (23.3)				11 (18.3)				
**WHOQOL**	**Total (points)**	46.4	18.2	44.5	12.9	52.6	18.9	53.3	14.4	**< 0.001**^1^
	Psychological domain	29.3	16.9	31.0	12.5	37.7	19.2	38.0	17.0	**< 0.001**^1^
	Physical domain	46.7	21.6	50.0	12.5	53.2	21.9	56.0	14.0	**< 0.001**^1^
	Social relations	59.2	22.7	56.0	12.5	63.9	22.7	72.0	15.5	**< 0.001**^1^
	Functioning in the environment	50.6	23.3	50.0	17.3	55.6	23.4	56.0	17.0	**< 0.001**^1^

Abbreviation: *SD* standard deviation, *Me* median, *Q* quartile deviation

^1^Wilcoxon test

**Fig. 2:**
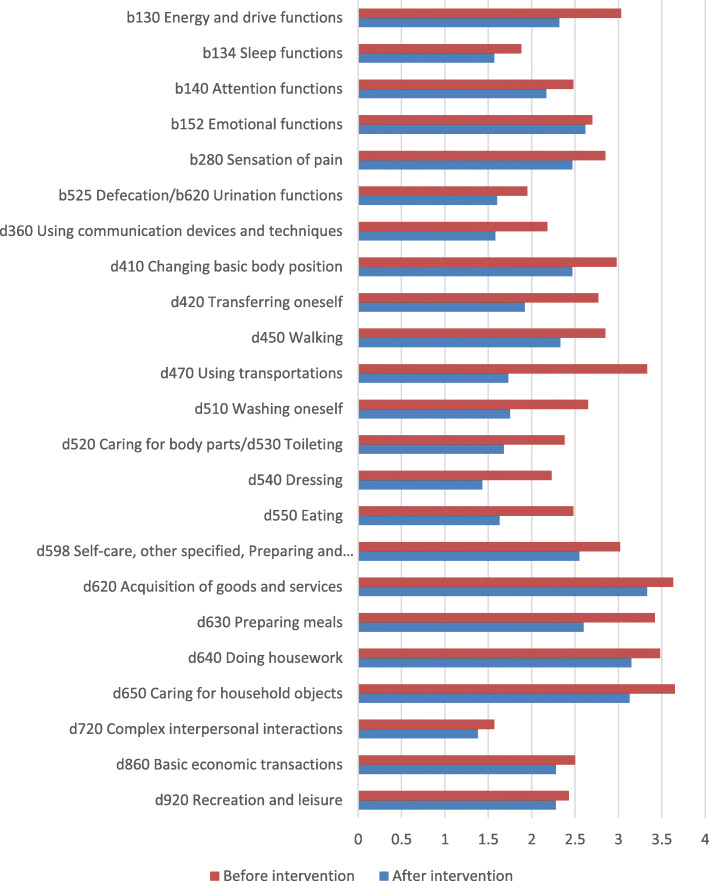
Health information linked to the ICF

In order to enable comparability of collected data worldwide, the selected measurement parameters were recorded graphically and numerically in terms of function, activity, and participation by the use of codes and the average score of ICF qualifiers for individual categories (Fig. 2). With regard to a description of the patient’s situation, 25 ICF codes were used, including six describing body functions and 19 describing activity and participation. A graphic presentation of the data in the form of qualifiers allows researchers to observe the condition of the patients before and after the physiotherapy program.

## Discussion

Hospice care is an extremely important element of the modern healthcare system. Owing to the development of science and medicine, the life expectancy of patients with severe chronic diseases is extended, and the moment of death is postponed [17]. Hospice care, understood as a comprehensive set of medical and rehabilitation services, is a relatively new solution, while, apart from controlling pain and other symptoms related to the disease, it is also important to improve the functioning and quality of patients’ lives. Physiotherapy is becoming a very important component of hospice care [38].

The average functional level of ADL and IADL of the examined patients before the beginning of physiotherapy in the home was low. There was a high average pain intensity and a high risk of falling. Moreover, a high incidence of depression and a poor quality of life, especially in the psychological domain, were recorded. The population of palliative patients was diverse regarding functional performance; some patients were still able to cope with some daily activities, while others were very weak. After the completion of the intervention program, a statistically significant improvement was observed in the patients in the many areas investigated in particular, in the scope of performing basic everyday activities, mobility, and balance. Moreover, a reduction in the level of depression and an improvement in the quality of life were noted. Patients were still diverse in functional status, and their condition in many cases still indicated severe restrictions, but it was better than before the program. It is worth emphasizing that each sign of improvement, even the slightest one, is very important for patients with severe chronic or fatal diseases.

The primary objective of our physiotherapy program was to improve daily activities for patients in the home receiving hospice services. The average level of ADL in the group we studied in the pre-test was low (mean 2.9). The most common problem before starting the physiotherapy program was a lack of independence in bathing and showering (73.3%) and transferring -functional mobility (61.7%). Other activities that involved the greatest, and even total, restrictions were dressing (55.0%) and toilet hygiene (55.0%; getting to the toilet, cleaning oneself, and getting back up). After the intervention, we observed a significant reduction in the frequency of dependence in almost all researched basic activities. The lack of independence in bathing and showering decreased statistically significantly (*p* = 0.002), but most of the studied people still needed help in performing this activity (53.3%). After the intervention, the frequency of dependence in transferring decreased by half (to 30.0%; *p* < 0.001), which was very important for improving the performance of other basic activities, such as dressing and the possibility of using the toilet. The results obtained in the program are crucial, due to the fact that improving ADL is an important factor in delaying death and improving quality of life [39]. Park et al. observed that ADL was an important predictor of mortality in elderly people with a severe disease. Observation of individuals in hospice not subjected to rehabilitation interventions showed a more dynamic decline in function and approaching death the faster, the worse the functional state of the patient [40].

In our study, before starting the physiotherapy program, a low average test score was found when assessing gait, balance, and risk of falling (mean Tinetti POMA Scale = 8.2 points). Seriously ill palliative patients who have long periods of lying down, often after a surgery or other interventional treatment, have problems with gait and balance. In our study, after completing the intervention, in which gait and balance exercises were an important element, a significant mean improvement in gait and balance (*p* < 0.001) and risk of falling (mean Tinetti POMA Scale = 12.3; *p* < 0.001) was observed in the study group. There was a decrease in the number of patients with a high risk of falling. Protas et al. carried out an individualized balance and gait training program, according to the data identified from the POMA in residents of a nursing home. After 4 weeks of exercise (20 sessions), the researchers recorded an improvement in balance scores (respectively, the mean initial and final balance scores were 8.5, SD = 3.8 and 10.8, SD = 3.4) and in gait scores (6.7, SD = 2.3 initially and 7.7, SD = 2.0 finally) (*p* < 0.001) [41]. Falls are a special problem in patients in a hospice. Studies have shown that 50% of patients fall during the subsequent 6-month time frame. The effects of falls of hospitalized patients are related to lowering the functional quality of life, as well as increasing anxiety, pain, and suffering. Only a few studies and interventions have been carried out in this patient group. However, a substantial majority of all of the studies performed thus far have shown the benefits of physiotherapeutic interventions in the area of improving fitness, including functional mobility and balance [42].

The possibility to control a stable body posture is the basis for independent mobility and it is extremely important to performing daily activities [43]. In our study, very low IADL scores before the intervention (mean IADL = 11.9) were recorded. In the pre-test, virtually all complex IADL activities were a problem for most patients (from 63.3%, i.e., using the telephone, to 96.7%, i.e., shopping). In the post-test study, a improvement (*p* < 0.001) in the average IADL level (13.9) was observed. A improvement was noticed in the scope of moving further than walking distance (*p* = 0.023) and was associated primarily with the possibility of traveling with a caregiver to a health center. Other areas of improvement were: the possibility of preparing meals (*p* = 0.023), taking prescribed medications (*p* = 0.041), and managing money (*p* = 0.041). In the examined group, despite the observed significant changes, IADL performance was still a very high problem. Neo et al., in their meta-analysis referring to disability in the performance of daily activities by older people with cancer, emphasize the need for rehabilitation focused on functional independence, in connection with the finding of a significant frequency of restrictions in ADL and IADL [44].

Before starting the improvement program, we noted a high average pain intensity (5.8). After the intervention, a significant reduction (*p* < 0.001) in pain intensity was observed (5.1), although it remained mainly at a moderate level. Chronic pain is one of the major causes of disability in daily activities [45]. Pyszora et al., in a randomized controlled trial (RCT) in palliative patients with advanced cancer, conducted 12 physiotherapy sessions, including active exercises, myofascial release, and proprioceptive neuromuscular facilitation (PNF) techniques that result in significant pain relief [46]. Coelho et al. found that combining different methods in the treatment of pain could be promising [47]. Physical therapy modalities and rehabilitation techniques are important options in pain therapy [48–50].

In the pre-test, we observed the occurrence of mild or deep depression in all patients. At the end of the program, we noticed a improvement (*p* = 0.012) in the average GDS score, and the number of people with deep depression slightly decreased. Pyszora et al., in an RCT, achieved a significant reduction in fatigue and depression and an improvement in the general well-being of patients in physiotherapy [46]. Mishra et al., in a systematic review assessing the effectiveness of physiotherapeutic interventions in cancer patients, found a decrease in depression, fatigue, and sleep disorders, as well as improved quality of life, especially emotional well-being [51].

The emotional and functional states are associated with the sense and assessment of quality of life [52]. Before starting the physiotherapy program, a low overall quality of life was observed in the examined patients (mean 46.4), especially in the psychological field (mean 29.3). After completing the intervention, a statistically significant (*p* < 0.001) improvement in the overall quality of life and the quality of life in each of the areas was found. Similarly, Oldervoll et al., after a 6-week rehabilitation program in cancer patients, noticed a significant improvement in the quality of life in the emotional (*p* = 0.002) and social (*p* = 0.008) areas [53]. An important goal of palliative care is to improve the quality of life of patients and their families/carers who are facing the challenges of life-limiting disease.

When reviewing the scientific literature, especially in the area of interventions carried out in hospice patients, it can be noted that there is a small number of studies conducted on this topic, but the tools used there are very diverse. Individuals in hospice are a specific study group in which it is necessary to limit the number of measurements performed due to their health condition. This means that the results of studies performed by different authors cannot be compared. That is why we used the ICF in accordance with the WHO guidelines in our physiotherapy program. The International Classification of Functioning, Disability and Health (ICF) of the World Health Organization provides a normalized, standard language and a comprehensive framework for describing health and health-related conditions. The deployment of qualifiers consistent with the ICF scale for the assessment of individual categories assigned to individual items of the ADL and IADL scales allowed the researchers to evaluate responsively the problem before rehabilitation, and observed a significant improvement in case of the overwhelming majority of patients from a total dependency (qualifier 4) toward significant problems (qualifier 3). This meant that a completely dependent patient was able to participate actively in the performance of these activities, which greatly facilitates the care and nursing of carers. What is more, in most cases, improvement was even greater (qualifier 2), which meant that the patient required assistance in carrying out the activity rather than performing it instead of him (which may need a total assistance). Also, in many cases, it was possible to achieve even a return to full (qualifier 0) or almost full independence (qualifier 1). Therefore, it is worth mentioning that the use of the ICF scale to assess individual items of the ADL and IADL scales also let the researchers specify in detail which activities required care and which only assistance, as well as which of them the patient was able to perform alone without the help of another person. A very important advantage of ICF is the ability to present results in various areas of human functioning in a universal language, which gave the researchers the opportunity to compare data using different measuring tools.

### Limitations

The main limitation of our study was the lack of a control group and a random selection of the subjects for the study group, but in the situation of working with patients in hospice where time plays a very important role, we found it ethical to provide support at the same time to all patients wishing to participate in our research program during its duration.

## Conclusion

Taking everything into consideration, we proved a positive impact of a multi-component, individualized physiotherapy program on the functional performance and quality of life of individuals in hospice staying at home. Speaking of a modern approach to palliative and hospice care, apart from drug treatment and nursing care, rehabilitation should also play an important role, with particular emphasis on physiotherapy. Due to modern forms of physiotherapy, it is possible to adapt therapeutic methods to the individual needs and clinical condition of the patient [54]. Owing to the dynamics of population aging and the increase in the number of severe chronic diseases, including cancer, there is an urgent need to conduct more intervention studies in the group of patients in older age aimed at improving functional performance and comfort of life in this period of their life.

In conclusion:

1. The intervention implemented by a multi-component, individualized physiotherapy program, including, among others breathing, functional, transfer/mobility, balance, and relaxation exercises, as well as learning to use mobility aids and elements of the environment to move more efficiently, after 12 sessions with patients under hospice care at home, had a significant impact on the decrease of pain and the improvement of patients’ mobility, balance, and functioning in carrying out everyday activities.
2. The implementation of the physiotherapy program improved the emotional state and quality of life of patients receiving hospice services in the home.
3. The physiotherapy model tested in our study can be used as a model of good clinical practice in patients receiving hospice care, especially in those with better prognosis regarding survival time.
4. The results of our research provide evidence of the growing need for physiotherapy in individuals in hospice on the same level as medical and social care, as well as the continuation of larger-scale research aimed at preparing and verifying new physiotherapy protocols.
5. The use of a universal language to assess the condition of individuals in hospice, namely the ICF codes, will allow researchers to compare their investigation results.
6. It is really important to conduct further clinical research on the positive impact of various physiotherapy programs on the functional status and quality of life of patients referred to the hospice.

## Data Availability

The datasets used and analysed during the current study are available in Repositorium of University of Rzeszow. https://repozytorium.ur.edu.pl/handle/item/5093

## Abbreviations

WHO: World Health Organization
ICF: International Classification of Functioning, Disability and Health
ADL: Activities of Daily Living
IADL: Instrumental Activities of Daily Living
WHOQOL: World Health Organization Quality of Life
VAS: Visual Analogue Scale
GDS: Geriatric Depression Scale
AMTS: Abbreviated mental test score
